# A Minimal Model for the Mitochondrial Rapid Mode of Ca^2+^ Uptake Mechanism

**DOI:** 10.1371/journal.pone.0021324

**Published:** 2011-06-23

**Authors:** Jason N. Bazil, Ranjan K. Dash

**Affiliations:** Biotechnology and Bioengineering Center and Department of Physiology, Medical College of Wisconsin, Milwaukee, Wisconsin, United States of America; University of Maribor, Slovenia

## Abstract

Mitochondria possess a remarkable ability to rapidly accumulate and sequester Ca^2+^. One of the mechanisms responsible for this ability is believed to be the rapid mode (RaM) of Ca^2+^ uptake. Despite the existence of many models of mitochondrial Ca^2+^ dynamics, very few consider RaM as a potential mechanism that regulates mitochondrial Ca^2+^ dynamics. To fill this gap, a novel mathematical model of the RaM mechanism is developed herein. The model is able to simulate the available experimental data of rapid Ca^2+^ uptake in isolated mitochondria from both chicken heart and rat liver tissues with good fidelity. The mechanism is based on Ca^2+^ binding to an external trigger site(s) and initiating a brief transient of high Ca^2+^ conductivity. It then quickly switches to an inhibited, zero-conductive state until the external Ca^2+^ level is dropped below a critical value (∼100–150 nM). RaM's Ca^2+^- and time-dependent properties make it a unique Ca^2+^ transporter that may be an important means by which mitochondria take up Ca^2+^ *in situ* and help enable mitochondria to decode cytosolic Ca^2+^ signals. Integrating the developed RaM model into existing models of mitochondrial Ca^2+^ dynamics will help elucidate the physiological role that this unique mechanism plays in mitochondrial Ca^2+^-homeostasis and bioenergetics.

## Introduction

Calcium plays many key roles with respect to mitochondrial bioenergetics [1], [2], [3]. The currently accepted paradigm is that Ca^2+^ directly modulates mitochondrial NADH levels via allosteric activation of matrix dehydrogenases [4], [5], [6], [7], enhances ATP production via F_1_F_O_ -ATPase activation [8], [9], as well as, triggers a catastrophic phenomenon known as mitochondrial permeability transition [10], [11], [12]. Despite the causal nature of mitochondrial Ca^2+^, the mode of entry and exit remains relatively ill-defined. The general consensus is that a highly-selective, high-conductance Ca^2+^ channel, the Ca^2+^ uniporter (CU), accommodates Ca^2+^ entry while the combined action of an nH^+^/Ca^2+^ and an nNa^+^/Ca^2+^ exchanger facilitate Ca^2+^ removal from the mitochondrial matrix [13]. Recent findings have expanded this list of Ca^2+^ transport mechanisms to include the rapid mode (RaM) of Ca^2+^ uptake and the mitochondrial ryanodine receptor [14], [15], [16]. In fact, these two additional uptake mechanisms have been proposed to be alternative conformational states of the CU [3], [17], and quite possibly the permeability transition pore [13]. Of all the potential Ca^2+^ transport related proteins, the molecular identity of only two transporters, nH^+^/Ca^2+^ (letm1) and nNa^+^/Ca^2+^ (NCLX), have been recently discovered [18], [19]. Although it is strongly believed that the CU has also been identified (MICU1) [20], more conclusive evidence will be necessary to demonstrate its true identity and connect it to the Ca^2+^ channel identified in recent patch clamp experiments [21]. Nevertheless, much remains to be learned about the mitochondrial Ca^2+^ transport molecular machinery, and even more so, its role in Ca^2+^-induced mitochondrial dysfunction.

Mitochondria are faced with a multitude of cytosolic Ca^2+^ signals consisting of either low impulse, long duration or high impulse, short duration transients [22], [23], [24]. These signals have profound influence over the Ca^2+^-sensitive pathways that exist in mitochondria. In fact, mitochondria themselves are able to modulate and shape these cytosolic Ca^2+^ transients due to their uptake and sequestration capabilities [25], [26], [27], [28], [29]. Interestingly, the primary reputed Ca^2+^ uptake pathway, the CU, is only operational when Ca^2+^ rises in the µM range in the presence of physiological concentrations of Mg^2+^ [30]. Recent modeling efforts have demonstrated that the CU affinity for Ca^2+^ varies from 10 to 90 µM as Mg^2+^ levels rise from 0 to 5 mM [31], [32]. This low affinity for Ca^2+^ would appear to prevent the CU from facilitating any significant Ca^2+^ uptake when Ca^2+^ is in the physiological nM range. Thus, it seems that the RaM mechanism may be a component of the physiological response to upstream Ca^2+^ signaling. Supporting this view, many groups have observed mitochondria *in situ* exhibit RaM-like behavior when exposed to nM levels of Ca^2+^ [15], [24], [33], [34].

It must be stressed that *in situ*, the mitochondrial network contains contact points with the sarcoplasmic reticulum known as micro-domains [35], [36]. There is very strong structural evidence for the existence of these micro-domains [37] which is also supported by some mathematical modeling [38], [39]. It is believed that [Ca^2+^] rises in these micro-domains above 10 µM during the peak of the cytosolic Ca^2+^ transient. This helps explain the rapid rise in mitochondrial Ca^2+^ observed *in situ*; however, it fails to explain how isolated mitochondria are able to rapidly take up Ca^2+^ in the sub-micromolar range [40], [41], [42], [43]. When mitochondria are isolated and removed from their native environment, these micro-domains do not exist [36]. Moreover, the RaM mechanism helps explain the experimental evidence regarding the frequency modulated response to cytosolic Ca^2+^ pulses resulting in a more efficient maintenance of NADH/NAD levels in rat hepatocyte mitochondria [44]. In this work, Hajnoczky et al. conclude that the frequency modulation is more important than amplitude modulation with respect to Ca^2+^ signal transduction and bioenergetics. The response to each pulse resulted in a rapid increase in matrix Ca^2+^ via a RaM-like process. Thus, it is likely that both, micro-domains and RaM, help modulate mitochondrial bioenergetics and Ca^2+^-buffering in a tissue dependent manner.

Despite the large body of evidence suggesting that mitochondria are able to rapidly take up and buffer exogenous Ca^2+^, the traditional CU has been the primary Ca^2+^ uptake mechanism encoded in mitochondria-related mathematical models [31], [45], [46], [47], [48], [49]. Of these models, Marhl's group was the only one to specifically address this fast uptake mechanism for their whole cell model, although in a phenomenological manner [48], [49]. In a detailed non-linear dynamics study, they found that this fast component was important with respect to induced and sustained cellular Ca^2+^ oscillations [50], [51], but the bioenergetic consequences of these oscillations have yet to be systematically determined. Thus, the predictive power of these models regarding the link between Ca^2+^ and bioenergetics remains speculative. Considering the relative importance of Ca^2+^ with respect to mitochondrial metabolism and integrity, a more accurate model of Ca^2+^ sequestration including the RaM uptake pathway would help drive innovative therapies targeted at Ca^2+^-related mitochondrial dysfunction. Also, the manner in which mitochondria decode the cytosolic Ca^2+^ signals may prove pivotal in understanding the regulatory impact that Ca^2+^ possesses on cellular and mitochondrial processes.

Presented herein is a simple 4-state model that is capable of capturing the salient features of RaM. The model reproduces the available experimental data of rapid Ca^2+^ uptake in isolated mitochondria from both liver and heart tissues with good fidelity [42], [43]. The model is robust in its structure and is easily extendable to include more detailed regulatory components. We believe that the RaM mechanism proposed herein will serve as a means in which to help further explore the role Ca^2+^ plays in mitochondrial bioenergetics using mathematical modeling.

## Results

The model parameters that were estimated for the 4-state RaM model in order to reproduce the experimental data [42], [43] are presented in Table 1. The details regarding the parameter estimation and analysis are given in the Material and Methods. In brief, some of the model parameters were assumed to be identical irrespective of which RaM model (heart versus liver) was used to reproduce the experimental data in order to minimize the number of unidentifiable parameters. These parameters were identified during the model development stage. Specifically, each model shares the same parameter values for the RaM complex Ca^2+^ affinity, Ca^2+^ cooperativity, Ca^2+^ binding constant for state *I_1_* and state *O*-*I_1_* transition rate. When more data becomes available, it may be possible to identify these parameters with more confidence.

**Table 1:** 4-State RaM Model Parameters.

Parameter	Definition	Value (95% Confidence Interval)		Units
		Heart	Liver	
*X_RaM_*	RaM Activity	0.0551 (0.054–0.056)	0.0126 (0.012–0.013)	nmol mg^−1^s^−1^
*K_Ca_*	RaM Complex Ca^2+^ Affinity	340 (331–348)	340 (331–348)	nM
*n*	Ca^2+^ Cooperativity	24	24	unitless^a^
*K_O_*	State *O* Ca^2+^ Binding Constant	224 (223–224)	196 (195–196)	nM
*K_I_*	State *I_1_* Ca^2+^ Binding Constant	110 (109–110)	110 (109–110)	nM
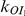	State *O*-*I_1_* Transition Rate	100	100	s^−1^ b
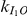	State *I_1_*-*O* Transition Rate	0.0364	0.009	s^−1^ c
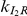	State *I_2_*-*R* Transition Rate	0.0384 (0.0346–0.0423)	100^a^	s^−1^
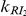	State *R*-*I_2_* Transition Rate	3.98×10^−6^ (3.98×10^−9^–1.27×10^−3^)^d^	1^a^	s^−1^

a Removed from confidence interval analysis due to unidentifiability. In this case, during the confidence interval estimations, the parameters were fixed to their values as shown.

b Fixed to the upper bound time constant of RaM in the open state [14].

c Set by enforcing microscopic reversibility where 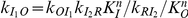.

d When the estimated lower bound was less than 0, it was set to 10^−3^ of the best parameter value.

**Figure 1:**
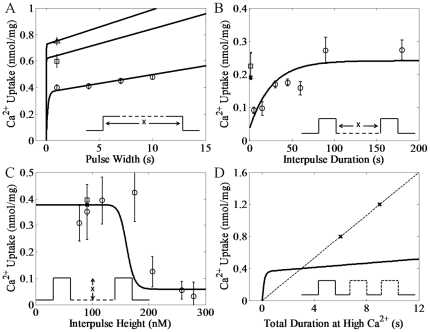
Simulations of the experimental data of Ca^2**+**^ uptake facilitated by RaM in heart. The corresponding pulse waveform for each experiment is shown in each panel. A) The model reproduced the measured Ca^2+^ uptake responses to pulses of Ca^2+^ at varying pulse heights and durations. The circles represent the Ca^2+^ uptake from a single pulse at a pulse height of 201 nM with an increasing pulse duration. The square and triangle represent the Ca^2+^ uptake from a 1 s duration Ca^2+^ pulse at a pulse height of 567 and 934 nM, respectively. The prepulse height for each pulse was 53 nM. B) The model reproduced the measured Ca^2+^ uptake response to a second pulse of Ca^2+^ following a previous pulse of the same magnitude at varying interpulse durations. The circles represent the Ca^2+^ uptake from a second pulse immediately after and up to 180 s after the first pulse. Both pulses were 5 s in duration at a pulse height of 209 nM with an interpulse height of 98 nM. The square represents a single, 5 s pulse at a pulse height of 181 nM with a prepulse height of 86 nM. C) The model reproduced the measured Ca^2+^ uptake response to a second pulse of Ca^2+^ following a previous pulse of the same magnitude at varying interpulse heights at a fixed interpulse duration of 60 s. Each pulse duration was 5 s. The circles represent the Ca^2+^ uptake from the second pulse at a pulse height of 274 nM. The square represents a single pulse at a pulse height of 273 nM for a duration of 5 s. D) The model reproduced the enhanced Ca^2+^ uptake response to multiple, short pulses versus a single, long pulse. The dashed line follows the trend of increasing Ca^2+^ uptake for in an increasing the number of pulses. All simulation results are either shown as x's or a solid line depending on the nature of the simulation.

**Figure 2 pone-0021324-g002:**
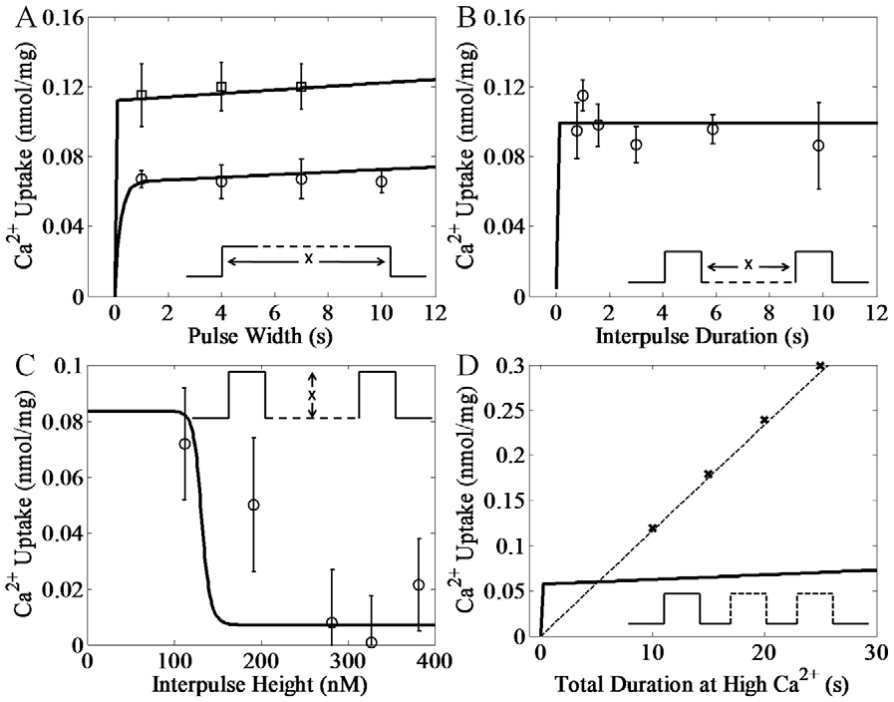
Simulations of the experimental data of Ca^2**+**^ uptake facilitated by RaM in liver. The corresponding pulse waveform for each experiment is shown in each panel. A) The model reproduced the measured Ca^2+^ uptake responses to pulses of Ca^2+^ at varying pulse heights and durations. The circles and squares represent the Ca^2+^ uptake from pulses at pulse heights of 171 and 281 nM, respectively, with an increasing pulse duration. B) The model reproduced the measured Ca^2+^ uptake response to a second pulse of Ca^2+^ following a previous pulse of the same magnitude at varying interpulse durations. The circles represent the Ca^2+^ uptake from a second pulse immediately after and up to 10 seconds after the first pulse. Both pulses were 5 s in duration at a pulse height of 421 nM with an interpulse height of 50 nM. C) The model reproduced the measured Ca^2+^ uptake response to a second pulse of Ca^2+^ following a previous pulse of the same magnitude at varying interpulse heights at a fixed interpulse duration of 1 second. The circles represent the Ca^2+^ uptake from the second pulse at a pulse height of 480 nM. Each pulse duration was 10 s. The Ca^2+^ uptake values were adjusted to compensate for CU activity to set the minimum uptake to be zero. D) The model reproduced the enhanced Ca^2+^ uptake response to multiple, short pulses versus a single, long pulse. The dashed line follows the trend of increasing Ca^2+^ uptake for in an increasing the number of pulses. All simulation results are either shown as x's or a solid line depending on the nature of the simulation. In all cases, the prepulse height for each pulse was near 50 nM.

Using the heart parameters listed in Table 1, the model simulations compared to the experimental data for heart RaM [42] is presented in Figure 1. The model was able to reproduce the Ca^2+^ uptake profiles for a sustained Ca^2+^ pulse (Figure 1A), varied interpulse durations (Figure 1B), varied interpulse heights (Figure 1C) and the increases in Ca^2+^ uptake efficiency for multiple pulses versus a single, sustained pulse (Figure 1D). The model was also able to simulate the liver experimental data for liver RaM [43] using the liver parameters listed in Table 1 as shown in Figure 2. Similar to the heart simulations, the model captured the Ca^2+^ uptake profiles for a sustained Ca^2+^ pulse (Figure 2A), varied interpulse durations (Figure 2B), varied interpulse heights (Figure 2C) and the increases in Ca^2+^ uptake efficiency for multiple pulses versus a single, sustained pulse (Figure 2D).

**Figure 3 pone-0021324-g003:**
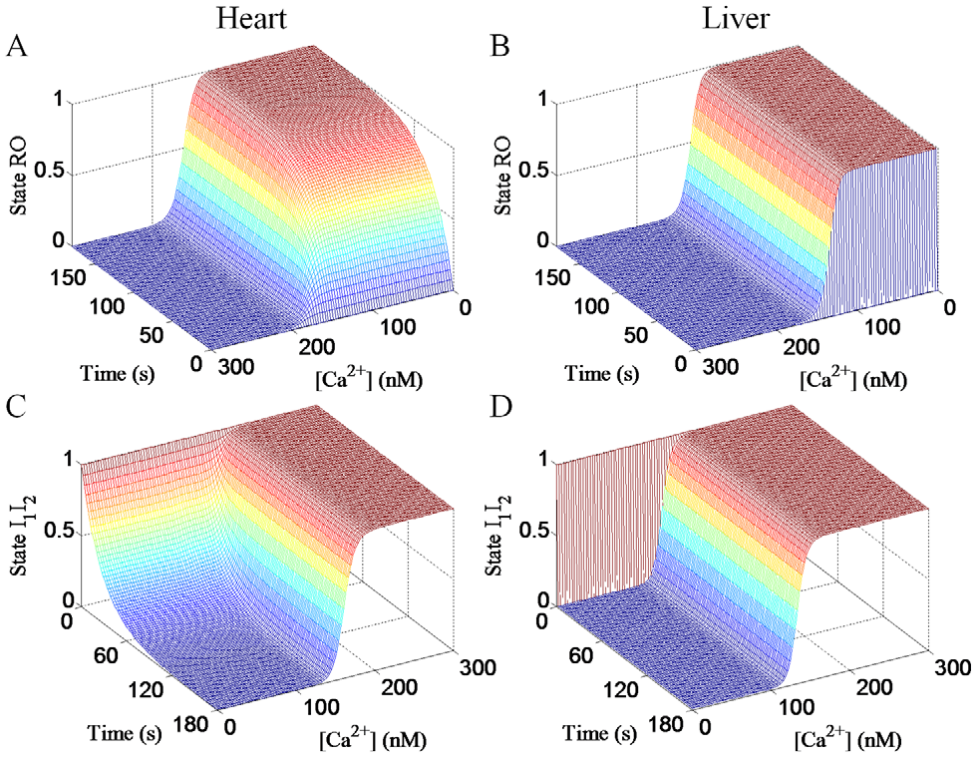
The major differences between the dynamics of the RaM model parameterized with the heart and liver parameters. The model was used to simulate the fraction of RaM in state *RO* and *I_1_I_2_* as a function of [Ca^2+^] and time to highlight the quantitative differences between heart and liver RaM as suggested by the available experimental data. Heart RaM only recovers back to the resting state when Ca^2+^ levels fall below 100–150 nM and sufficient time has passed (60–90 s). Liver RaM shows a very similar Ca^2+^-dependence; however, the time-dependence for recovery is much shorter (less than 1 s).

**Figure 4 pone-0021324-g004:**
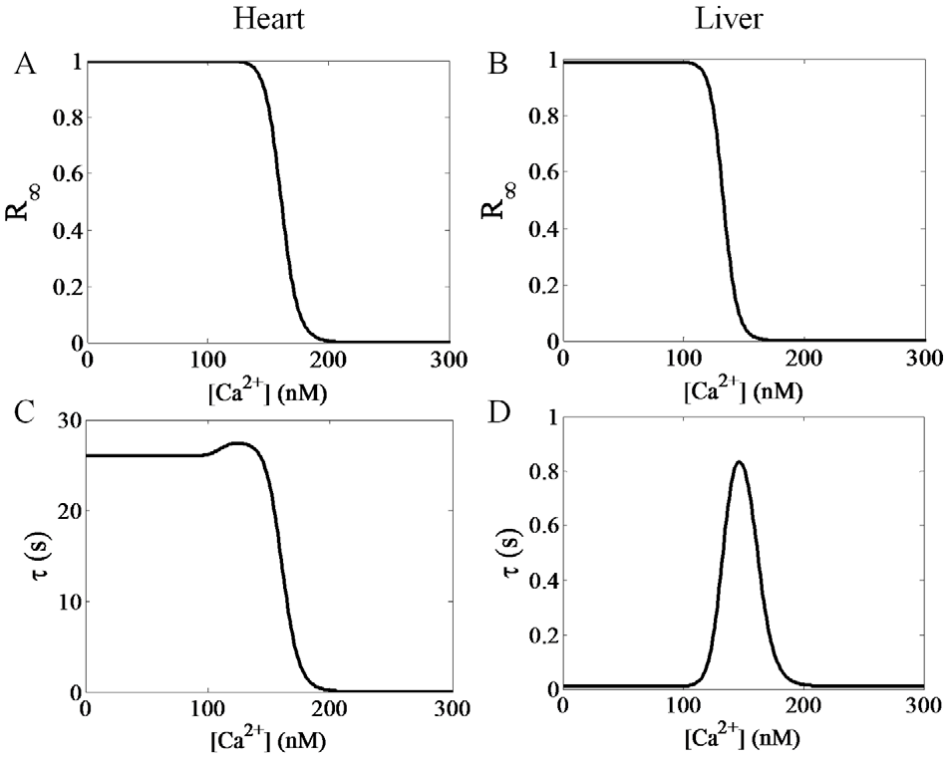
The steady state values for state *R* and time constants for the models as a function of Ca^2+^ is presented for heart and liver RaM. Although the steady state values for state *R* (A and B) are identical, the time constants (C and D) are dramatically different between heart and liver RaM.

Figures 3 and 4 show the major differences between the dynamics of the 4-state RaM model simulated with the heart parameters and the liver parameters. The Ca^2+^- and time-dependent recovery (3A and 3B) and inhibition (3C and 3D) profiles for heart and liver RaM demonstrate the major differences between the two RaM types. Although the steady state values for state *R* (4A and 4B) are identical, the time constants (4C and 4D) is dramatically different between heart and liver RaM. When [Ca^2+^] exceeds the *K_O_*, the time-dependent activation rates are identical; however, when [Ca^2+^] drops below the *K_I_*, the time-dependent recovery rates varies considerably between heart and liver RaM. For example, heart RaM takes up 90 seconds to recovery while liver RaM recovers in less than 1 second. Equations 4 and 1b were used to generate the surface plots for the recovery and inhibition profiles, respectively. Equations 5 and 6 were used to plot the time constants and the steady state values for state *R*, respectively.

**Figure 5:**
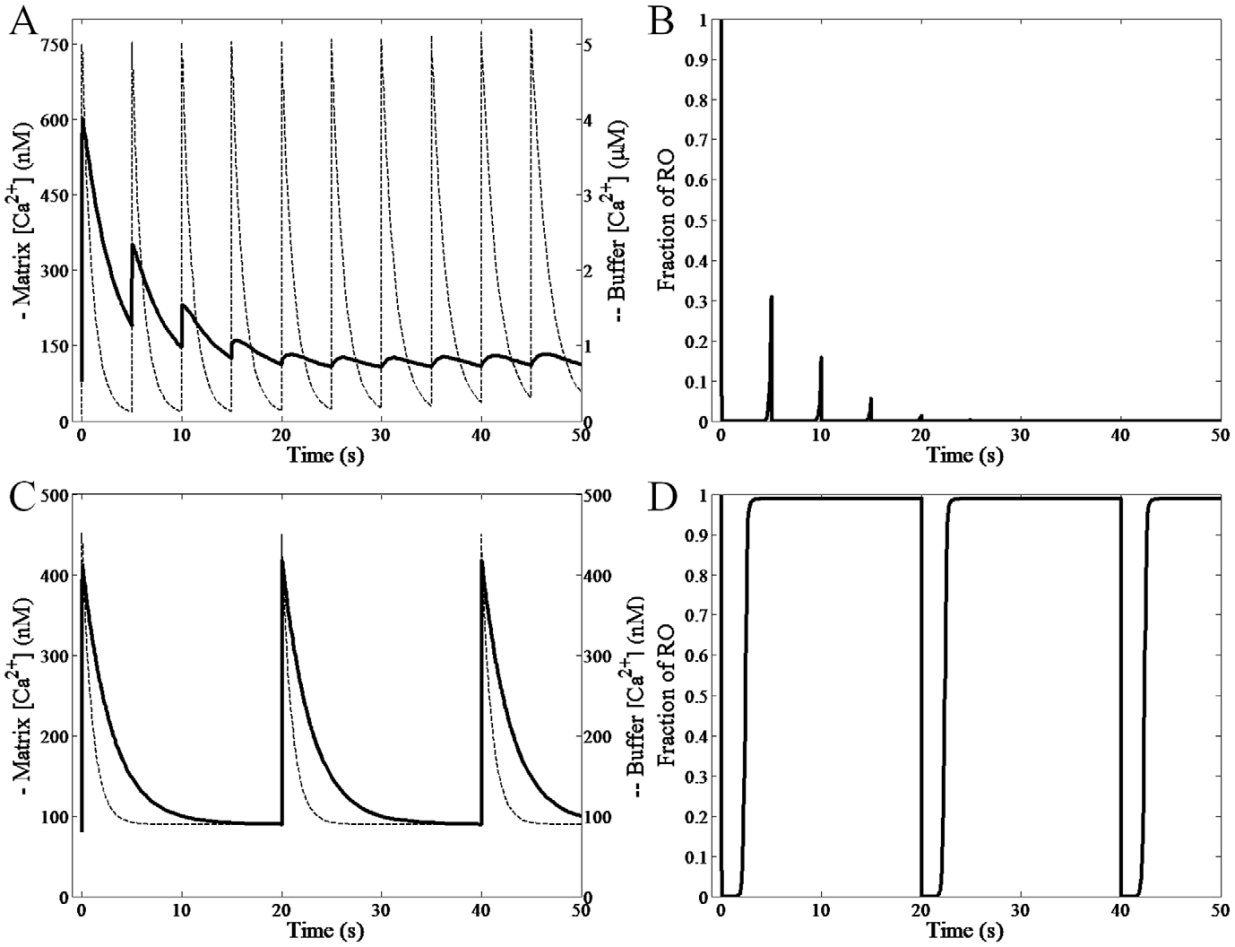
Corroboration simulations for the RaM model. The experimental conditions outlined in [14] were simulated using the extremely simplified Ca^2+^ model described in the Material and Methods. A) The matrix and buffer Ca^2+^ dynamics were simulated for the high amplitude and frequency experiment. B) The corresponding fraction of RaM in the *RO* state is shown. C) The matrix and buffer Ca^2+^ dynamics were simulated for the low amplitude and frequency experiment. B) The corresponding fraction of RaM in the *RO* state is shown.

To corroborate the RaM model, the experimental data presented in [14] was simulated. In these experiments, the liver mitochondrial Ca^2+^ uptake profiles were probed using Ca^2+^ pulses of varying amplitudes and frequencies. For details regarding the methodologies employed, see the Material and Methods section. The model results are presented in Figure 5 and reveal that the experimentally observed trends are reproduced quite well. Figure 5A demonstrates the model's ability to capture the attenuation of Ca^2+^ uptake facilitated by a gradual increase in buffer [Ca^2+^] (see Figure 2 in [14] for comparison). Figure 5B is a plot of the corresponding fraction of RaM in state *RO* and shows that as the pulse train continues, fewer and fewer RaM complexes recover due to Ca^2+^-induced inhibition. Figure 5C shows that when the frequency, as well as the intensity, of the pulses are reduced, the mitochondrion react to each pulse in a nearly identical fashion (see Figure 3 in [14] for comparison). Figure 5D shows that the corresponding fraction of RaM in state *RO* fully recovers between each pulse. It is interpreted that the pulses at a lower amplitude and frequency allow the local [Ca^2+^] in the buffer to diffuse away from the mitochondrion and thus enable RaM to reset before the next pulse is given. Although the simulations give concentration values for both extra-matrix and matrix Ca^2+^, these are merely approximated based of the available information presented in [14], and the results should only be interpreted as a *qualitative* corroboration of the uncalibrated data.

## Discussion

It is quite remarkable that the simple 4-state RaM model captured the salient features of RaM identified in the available experimental data sets for both heart and liver tissues. Specifically, it has been shown that i) when begun, Ca^2+^ uptake via RaM completes within 30 ms [52], ii) when resting Ca^2+^ levels exceed 200 nM, RaM uptake is minimal [42], [43], iii) to reestablish rapid Ca^2+^ uptake, the Ca^2+^ level must decrease below approximately 100–150 nM for a period of time [42], [43], iv) Ca^2+^ uptake via RaM is a saturable process [43], and different tissues possess characteristically distinct RaM mechanisms [42], [43]. The model reproduces each of these major features presented by RaM as shown in Figures 1–5.

Although the 4-state RaM models for the heart and liver are structurally identical, there were differences between the two parameter sets used to simulate their respective data sets. This is evident after inspection of the available experimental data [42], [43]. For the sustained Ca^2+^ pulse experiments, it's quite clear from the Ca^2+^ uptake versus time profile that, the Ca^2+^ uptake for liver RaM is quite flat for [Ca^2+^] below 300 nM, but a small “leak” is present for the heart RaM for [Ca^2+^] as low as 200 nM. Therefore, in order to simulate this leak phenomenon for heart RaM, the rate constant 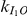 was required to be near 0.036 s^−1^ but needed to be below 0.01 s^−1^ to simulate the liver response (see Table 1). It's interesting to note that when the “slow” uptake rates are calculated from the slopes of the Ca^2+^ uptake profiles, they fall right on top of a curve defining the CU activity as a function of Ca^2+^ (not shown). Thus it appears likely that this small leak phenomenon for heart RaM is a manifestation of CU activity at low Ca^2+^. If the data is adjusted for this observation, 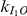 for heart RaM would be very near or equal to that of 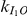 for liver RaM. Because the model is thermodynamically constrained via microscopic reversibility, reducing 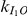 for heart RaM to match that for liver RaM would require 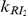 to increase to 1.65×10^−5^ s^−1^. This value is well within the given confidence interval shown in Table 1 and changing it as shown would have no impact on the model simulations. Another difference between the heart and liver RaM is revealed by the experimental results shown in Figures 1C and 2C. In these experiments a subtle difference between the two RaM types is observed. The RaM mechanism in liver tissue is activated at [Ca^2+^] above 196 nM while it takes more than 224 nM in heart tissue. This difference is very small, approximately 25 nM, and may be too small to experimentally confirm considering the uncertainty in the interpulse heights.

Buntinas et al. [42] report that there are at least two-types of RaM entities present in heart mitochondria based on ruthenium red titrations. One population is sensitive to ruthenium red staining, as with liver RaM, while the other is relatively insensitive to the staining. Perhaps the biphasic recovery exhibited by heart RaM due to the existence of two populations of RaM transporters with their own Ca^2+^- and time-dependencies. This concept is explored deeper in the File S1. For example, a 170 nM Ca^2+^ pulse yielded an uptake of Ca^2+^ of approximately 0.08 nmol/mg by liver RaM as in seen in Figure 2A, whereas a 200 nM Ca^2+^ pulse with an interpulse duration of less than 1 second resulted in an uptake of Ca^2+^ of 0.09 nmol/mg by heart RaM as seen in Figure 1B. It seems that a small fraction of RaM in heart tissue is liver-like with the majority possessing RaM characteristics identified as the heart-type. If this is indeed the case, the placement of the different types of RaM in specific regions of the cell (intermyofibrillar, perinuclear and subsarcolemmal) may prove critical in the response of mitochondria to cytosolic Ca^2+^ transients [53].

Corroboration is a necessary step in order to affirm the underlying hypotheses used to derive any model. This was done by the simulating experimental data presented by Gunter et al. [14]. In order to simulate the data presented in Figure 5, a very basic model of mitochondrial Ca^2+^ dynamics was developed. This basic model consisted of Ca^2+^ influx via RaM and the CU and efflux via the Ca^2+^/H^+^ exchanger. This approach was favored over a more encompassing endeavor because far fewer adjustable parameters were required; moreover, the experimental data was qualitative in nature and prevented any quantitative analysis. The model was driven by Ca^2+^ pulses generated with square waves, and despite the very simple dynamical model used, the simulations were able to capture the matrix Ca^2+^ trends very well. Namely, the simulation results were able to demonstrate that i) at high amplitude and frequency Ca^2+^ pulses, the mitochondrion was unable to respond fast enough due to the Ca^2+^-induced inhibition of the RaM complex and ii) pulses delivered at a lower amplitude and frequency produced a more robust response that enabled the mitochondrion to sequester more Ca^2+^. It follows that a more sophisticated modeling effort must follow and demonstrate how mitochondria respond to rapid changes in extra-mitochondrial Ca^2+^ when more quantitative data becomes available.

The degree of control required to assay RaM kinetics at a quantitative level is very demanding. It is highly likely that no matter how skillful the experimenter or precise the experiment, endogenous uncertainty may dominate the measured response and influence the parameter estimation. For example, the quantitative difference between liver and heart RaM could be attributed to the differences in their native environments. Perhaps cardiolipin or cardiac specific mitochondrial proteins are responsible for differences in rate constants between the heart and liver RaM parameters via direct interaction with the RaM complex [54]. Moreover, the quantitative differences could be attributed to different energetic states presented by the different mitochondrial types. Unfortunately, no quality control information was given with the experimental data such as a respiratory control index (RCI). If the RCI values were different for different mitochondrial preparations, some of the quantitative differences observed between and within the two data sets could be explained. Also, since the Ca^2+^ influx through RaM is very large, the mitochondria could have overcome the buffering capacity of the buffer for the low concentration Ca^2+^ pulses and dropped the [Ca^2+^] below the reported value. This would underestimate RaM's capacity for Ca^2+^ uptake and hinder model fitting to the reported experimental data. Despite any issues with the data, the underlying premise used to construct the model could be incorrect; however, considering that the cycling mechanism was able to produce all of the major features presented by the data, this scenario is unlikely.

**Figure 6:**
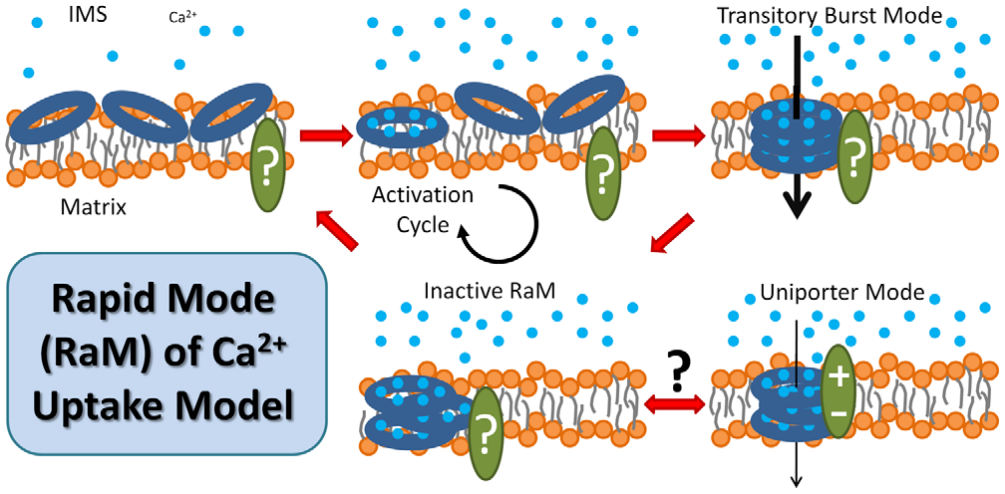
Schematic of the proposed model of the mitochondrial RaM Ca^2+^ uptake mechanism. The mechanism is based on that published by Starkov [13]. The model assumes that Ca^2+^ is required to initiate an activation cycle where the complex enters a transitory state of high Ca^2+^ conductance before entering an inactivated state. While the external Ca^2+^ remains elevated, the mechanism either stays locked in this quiescent state or triggers another conformational change eliciting the slow uptake (CU) mode. Once the external Ca^2+^ falls below a threshold concentration, the RaM complex dissociates and resets awaiting another trigger pulse of Ca^2+^.

Mathematical models are informative abstractions of a physical reality, and their development and subsequent predictive capabilities are strongly dependent on the availability of experimental data, more so than the underlying theoretical constructs in which they are based. Although the 4-state RaM model presented herein is able to reproduce the experimental data regarding the time- and Ca^2+^-dependencies of RaM dynamics for both liver and heart tissue very well, two data points shown in Figures 1C and 2C need addressed. These mismatches between model simulation and experimental data can be attributed to some apparent self-contradictory data presented in both data sets. For example, the data shown in Figure 6 of [42] reports Ca^2+^ uptake at “high calcium” which corresponds to approximately 140 nM Ca^2+^ according to the figure caption. However, in Figure 5 of the same data set , an interpulse concentration of 180 nM Ca^2+^ is sufficient for the RaM mechanism to fully reset and allow a full Ca^2+^ uptake cycle. It is unclear how 140 nM Ca^2+^ can trigger an activation cycle when the mechanism is still in the resting state at 180 nM. As another example, a single pulse of 484 nM Ca^2+^ shown in Figure 2 of [43] results in approximately 0.2 nmol/mg of Ca^2+^ uptake; however, in the same data set, a 421 and 400 nM Ca^2+^ pulse results in 0.1 nmol/mg of Ca^2+^ uptake as shown in Figure 4 and 6, respectively [43]. Moreover, it is unclear why 397 nM Ca^2+^ does not fully inhibit the RaM mechanism and prevent further Ca^2+^ uptake for a second pulse shown in Figure 5 of [43]. In another related example, the data point corresponding to 180 nM in Figure 1C is difficult to fit when the Ca^2+^ cooperativity constant is less than 60. If this value is increased, the model can simulate the Ca^2+^ uptake at 180 nM exceptionally well (not shown); however, the fits to the liver data set are compromised since both models share this parameter. A very similar, but opposite, reason exists why the model cannot reproduce the Ca^2+^ uptake corresponding to 200 nM in Figure 2C. A Ca^2+^ cooperativity constant less than 14 is necessary to fit this point. Perhaps, the RaM complex exhibits some sort of adaptivity property and/or a tissue-dependent degree of Ca^2+^ cooperativity which would help explain these apparent behavioral inconsistencies. However, until more quantitative data examining the intriguing properties of the RaM complex is collected, this possibility remains merely speculative. Regardless, the model is adequately able to capture the trends observed in both data sets.

The model was constructed assuming that there was no requirement for some form of exogenous energy input in order to cycle. However, some of the transition rates for the state model may in fact be voltage-dependent. This would bias the mechanism to cycle forward only in the presence of a sufficiently polarized membrane potential. In fact, it has been shown that the uncoupler, CCCP, abolishes any Ca^2+^ uptake [16]. This implies that RaM exhibits some form of voltage-dependence. We have addressed this dependence by considering that the Ca^2+^ uptake when RaM is in the open state is determined by the electrophoretic driving force alone (see below). One additional possible voltage-dependent mechanism is that the transition from one state to another is facilitated by the presence of a membrane potential. Unfortunately, the available experimental data do not allow testing of this hypothesis. Therefore, the model was constructed assuming that there was no requirement for an energy input; the transition rates were assumed to be voltage-independent. Future experiments will need to be carried out to facilitate an exploratory endeavor regarding these two different mechanisms. Considering the large body of evidence that the RaM is a precursor state of the CU [3], [17], and the evidence that the CU is a voltage-gated channel [55], it seems plausible that RaM possesses a similar voltage-dependence. Until the molecular identity of RaM is discovered, ascertaining the explicit voltage dependence of RaM will be difficult.

The functional form of the voltage-dependence of the Ca^2+^ transport expression (equation 7) warrants some discussion. Although we assume a simple, centered Eyring energy barrier, many other variants, such as a trapezoidal energy barrier or Goldman-like energy barrier, can fit the experimental data equally well. This is due to our assumption that ΔΨ was constant throughout the experimental conditions. Considering that the experimental protocols used saturating substrate to energize the mitochondria, this assumption is justified. During a Ca^2+^ pulse, the ΔΨ would be expected to depolarize a few mVs; however, unless [Ca^2+^] rises above 10 s of µM, the ETS capacity would maintain a steady ΔΨ. Therefore, in order to maintain identical fits to the data with a different voltage-dependence for the Ca^2+^ transport equation, only the activities (*X_RaM_*) would need to be adjusted. It's quite possible that the voltage-dependence of the Ca^2+^ transport via RaM is similar to that found for the CU [32]. Repeating the experiments in [42], [43] with a membrane potential titrating agent such as 2,4-dinitrolphenol, would help establish the voltage-dependence. Despite uncertainties regarding the voltage-dependence of the Ca^2+^ transport expression (equation 7), the underlying Ca^2+^-dependence and cycling mechanism remain valid.

The unique characteristics of RaM reveal some potential insight into the molecular identity of this obscure entity. One relatively overlooked potential molecular component of RaM is the rather conserved eukaryotic protein, p32/gC1qR [56]. The protein is synthesized with an N-terminal mitochondrial targeting sequence, and its amino acid sequence contains many aspartic and glutamic acid residues that are distributed on the surface of the protein [57]. Calsequestrin, a high capacity Ca^2+^-binding protein stored in the SR, also contains many aspartic and glutamic acid residues and is capable of binding up to 40–50 Ca^2+^ ions per molecule [58], [59]. Considering the similar features that these two proteins share (oligomer functionality, highly acidic with many aspartic and glutamic acid residues, a pI value near 4 and located in Ca^2+^ rich environments), it's possible that p32 may play a role in Ca^2+^ storage in the matrix or Ca^2+^ transport across the mitochondrial inner membrane. In the CU model proposed by Starkov, several p32 trimers are recruited in the inner membrane to form the CU [13]. In the context of the RaM model presented herein, the rather large Hill coefficient needed to fit the data, may represent the necessity of many Ca^2+^ ions to associate with p32 to form a functional oligomer that is capable of transporting Ca^2+^ down its electrochemical gradient across the mitochondrial inner membrane. Also, since the proposed mechanism is dependent on Ca^2+^ binding to the aspartic and glutamic acid residues, the pH dependence on Ca^2+^ uptake via the uniporter measured by Moreau et al. [60] indirectly supports the p32-based Ca^2+^ uptake model. Based on Starkov's original hypothesis, a plausible mechanism capable of explaining much of the observed RaM-like properties is depicted in Figure 6. It's quite feasible that the Ca^2+^-dependent activation cycle and transient burst of Ca^2+^ uptake implied from the available experimental data may be a result of the physical, “on demand” construction of the CU. In fact, we have successfully fit recent experimental data on matrix Ca^2+^ uptake when the buffer Ca^2+^ was in the sub-micromolar range [40] without ad hoc alterations to our CU model by assuming that RaM and the CU are indeed the same entity but in different conformational states (not shown). We are currently investigating the results and their potential implications. Although, this an intriguing hypothesis with some experimental support [61], it is still highly speculative, and proteomics must ultimately reveal the true identity of both the RaM complex and the CU.

In recent years, stochastic models have become increasingly prevalent when describing various biological phenomena [62], [63]. This is partly due to the inability of deterministic models to capture the heterogeneous response to a given stimuli for a variety of cellular processes ranging from excitation-contraction coupling in muscle [64] to T-cell receptor signaling [65]. Recently, Marhl's group has investigated the stochastic processes responsible for Ca^2+^ signaling in a variety of tissues [66], [67], [68]. This work built upon and confirmed theoretical predictions regarding the critical components regarding the stochastic events of single cells leading up to the seemingly deterministic response of whole organ systems [69], [70]. In light of the stochastic ensemble defining cellular Ca^2+^ dynamics, the RaM mechanism may be involved with the underlying events that comprise the global Ca^2+^ response. In fact, Gunter et al. originally proposed that the RaM mechanism is controlled by a stochastic process [71]. Considering the relatively few numbers of the CU (and by extension RaM) per mitochondrion [3], stochastic process may dominate the mitochondrial uptake dynamics in the sub-micromolar Ca^2+^ regime. Thus, a stochastic approach describing the RaM mechanism may serve as the natural evolution of the model.

In conclusion, we have presented a basic model of the recently uncovered RaM mechanism for Ca^2+^ uptake present in cardiac, liver and brain mitochondria. The model simulations are consistent with the available experimental data, and the model presents a unique opportunity to study the information encoded within the cytosolic Ca^2+^ transients presented to mitochondria *in situ*. We postulate that the frequency response of mitochondria is regulated by RaM and is critical to the translation of the cytosolic Ca^2+^ signal with respect to the cells energy supply and demand. We feel that the RaM model will serve as a means in which to further explore the role Ca^2+^ plays in mitochondrial bioenergetics *in situ* using mathematical modeling. Integrating this model into existing models of mitochondrial Ca^2+^ dynamics will help elucidate the physiological role this unique mechanism plays in mitochondrial Ca^2+^-homeostasis and bioenergetics.

## Materials and Methods

A quick overview of the available experimental data will first be discussed followed by the derivation of the 4-state RaM model. Although RaM is shown to be differentially regulated by a variety of effectors such as adenine nucleotides, spermine and ruthenium red, the primary goal of this work was to capture the time- and Ca^2+^-dependent dynamics of RaM in a mathematical formulation. Therefore, the models' ability to reproduce the data describing these dependences will be discussed herein. All calculations were performed with MATLAB® (2010a, 64-bit) on an Intel® Xeon® CPU(W3565 at 3.0 GHz) with 12 GB RAM. The results obtained were displayed using MATLAB®.

### Existing Experimental Data

**Figure 7:**
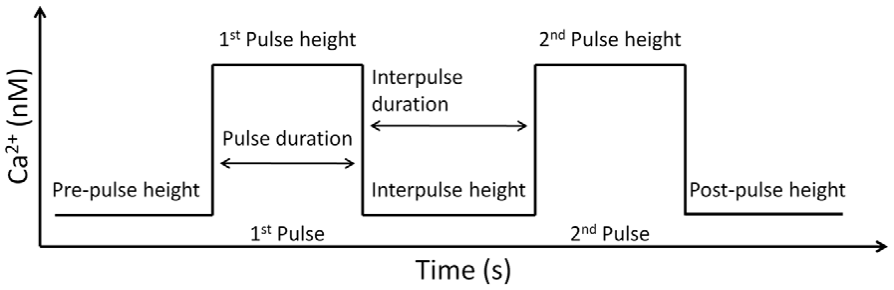
The Ca^2+^ pulse waveform used in the experimental setup. The Ca^2+^ pulse delivery system was capable of adjusting the pre-pulse height, pulse height, pulse duration, interpulse duration, interpulse height and post-pulse height. For details concerning specific operational characteristics of the apparatus, see [72].

The qualitative and quantitative characteristics of RaM were gathered from the literature [42], [43] where isolated Sprague-Dawley rat liver and White Leghorn chicken heart mitochondria were used. The experimental medium was similar for both types of mitochondria and consisted of 150 mM KCl, 24 mM K-HEPES, 0.1 mM KPi, 5 mM K-succinate and 0.1 mM sucrose. For the isolated heart mitochondrial experiments, 7.5 µM TPP^+^ was included in the experimental medium to inhibit the Na^+^-dependent Ca^2+^ extrusion pathway. A dual pipetter system was constructed that was capable of delivering repeatable Ca^2+^ pulses via the application of various Ca^2+^-EGTA/EDTA buffers [72]. For clarity, a generalized Ca^2+^ pulse waveform used in the experimental setup is presented in Figure 7. The Ca^2+^ uptake via RaM was quantified using radiolabeling, fluorescence and atomic absorption techniques, and in some cases, corrections were made to compensate for the Ca^2+^ egress via the Na^+^-independent Ca^2+^ efflux pathway. The time- and Ca^2+^-dependences of RaM facilitated Ca^2+^ uptake was quantified by adjusting the pulsatile Ca^2+^ input delivered to the mitochondrial suspension. The pulses were adjusted to either be rapid in succession, high in amplitude, possess high interpulse Ca^2+^ concentrations or a mixture of either (Figure 7). With this type of input, the experimental system response was sufficiently teased out at a quantitative level to facilitate the construction of a basic RaM model.

**Figure 8:**
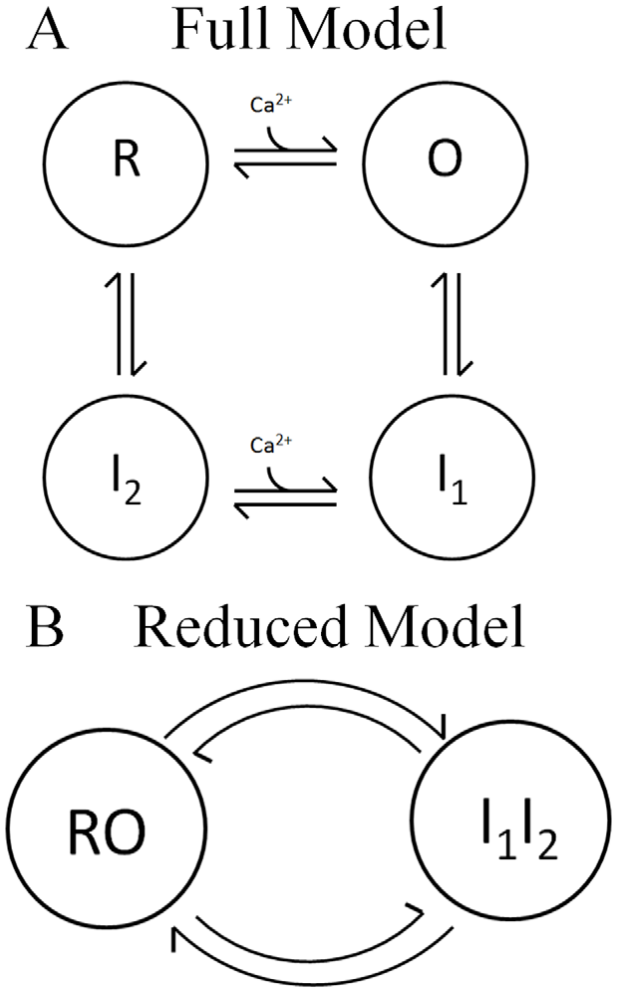
The state model used to derive the 4-state RaM model. A) The full 4-state model is pictured where *R*, *O*, *I_1_* and *I_2_* represent the resting state, open state, 1st inhibited state and 2nd inhibited state, respectively. B) For the reduced form, it is assumed that states *R* and *O* and *I_1_* and *I_2_* are in rapid equilibrium with each other. With this assumption, the 4-state model is reduced to a 2-state model where *RO* and *I_1_I_2_* represent the resting-open state and sum-total inhibited state, respectively. Applying conservation (i.e. *RO*+*I_1_I_2_* = 1), the 2-state model is further reduced to a single ordinary differential-algebraic equation.

The RaM model is developed here to reproduce the experimentally observed rapid Ca^2+^ uptake properties of mitochondria [42], [43] by invoking a suitable Ca^2+^ cycling mechanism. The proposed mechanism requires that Ca^2+^ binds to an external site(s) to initiate at very brief transient of high Ca^2+^-conductance that is indicative of RaM. After approximately 30 ms, the mechanism switches from this highly conductive state into an inhibited state. The mechanism is then locked into this inhibited state until the external Ca^2+^ is lowered below ∼100–150 nM. While the external Ca^2+^ lies below this value, the mechanism enters the resting state which takes up to 60–90 seconds for heart [42] but less than 1 second for liver [43]. Despite the 60–90 second reset time for heart RaM, a second pulse immediately following the first pulse uptakes nearly 25–30% of the initial pulse which suggests approximately a quarter of heart RaM resets on a similar time scale as liver RaM. Thus, it appears that heart RaM exhibits some sort of biphasic recovery. This biphasic recovery is addressed in the Discussion and File S1. The 4-state RaM model used to capture the experimental rapid Ca^2+^ uptake trends is pictured in Figure 8. The corresponding 5-state RaM model that was used to reproduce the biphasic recovery dynamics of the heart RaM is presented in the File S1. The rapid equilibrium assumption was used to reduce the full state models into their lower dimensional forms and to decrease the number of adjustable parameters. This assumption entails that the external Ca^2+^ binds to the external site(s) at least two orders of magnitude faster than the transition rates from the open/rest to inhibited states.

The RaM mechanism was modeled to adhere to the laws of thermodynamics. For example, the cyclical model obeys microscopic reversibility. The principle of microscopic reversibility, or detailed balance, has its origin in thermodynamics. For cyclical processes at equilibrium, the flow through each state in one direction must be equal to the flow in the opposite direction; otherwise, there would be no true equilibrium, and the system would require some form of energy input (i.e. electro- or chemical-potentials, ATP hydrolysis, etc.). Currently, there is no direct experimental evidence supporting the requirement of some form of energy input in order to cycle the RaM mechanism; therefore, the RaM model was constructed with microscopic reversibility strictly enforced. This removed a single adjustable parameter for each independent cycle from the optimization. If determined that the RaM mechanism is driven by some form of an energy input, the model can easily be refined to include such a dependence.

### 4-State RaM Model

To derive the 4-state RaM model shown in Figure 8, it is assumed that upon Ca^2+^ binding to the rest state (*R*), RaM enters the open state (*O*), and Ca^2+^ rapidly flows down its electrochemical gradient. Once open, RaM quickly closes and enters an inhibited state (*I_1_* and *I_2_*) until [Ca^2+^] is decreased below a threshold value. Applying the rapid equilibrium assumption for states *R* and *O* and *I_1_* and *I_2_*, the dynamical equations for the 4-state RaM model can be reduced to the following deterministic ordinary differential-algebraic equation
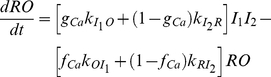
(1a)

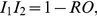
(1b)where *RO* represents the fraction of RaM in the rest and open states, *I_1_I_2_* is the fraction in the inhibited states, 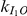 is the transition rate from *I_1_* to *O*, 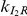 is the transition rate from *I_2_* to *R*, 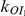 is the transition rate from *O* to *I_1_*, 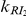 is the transition rate from *R* to *I_2_*, *f_Ca_* is the fraction of state *RO* with Ca^2+^ bound and *g_Ca_* is the fraction of state *I_1_I_2_* with Ca^2+^ bound. The relationship between the rest and open states as a function of Ca^2+^ is expressed as
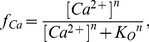
(2)where *K_O_* is the threshold concentration that triggers the cycling mechanism and *n* is the Hill coefficient representing the steepness of the profile for the fraction of bound versus free RaM. Therefore, the amount of RaM in the rest or open states can be computed using 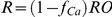 and 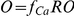, respectively. The relationship between the first and second inhibited states is similarly computed as
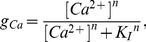
(3)where *K_I_* is the threshold concentration locking the cycling mechanism in the inhibited state, and *n* is the Hill coefficient representing the steepness of the profile for the fraction of bound versus free RaM for the inhibited states. The fraction of inhibited RaM without and with Ca^2+^ bound are computed as similar to the fraction of rest and open RaM states where 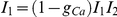 and 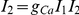, respectively.

The non-linear state model presented in equation 1 must be solved numerically unless the external [Ca^2+^] is constant. However, in all the experimental protocols [42], [43], the external [Ca^2+^] was held fixed. Thus, the non-linear state model simply becomes a linear state model, which can be integrated to obtain an analytical solution:
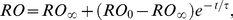
(4)where *τ* is the time constant for transitions between *RO* and *I_1_I_2_* at a given [Ca^2+^], *RO_∞_* is the steady state value of *RO* at a given [Ca^2+^] and *RO_0_* is the initial value of state *RO*. The time constant and the steady state value of *RO* at a given [Ca^2+^] are calculated using
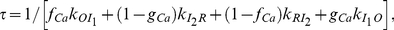
(5)and
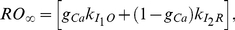
(6)


### Ca^2+^ Uptake Equation

With the dynamics of the 4-state RaM model known at any given [Ca^2+^], the Ca^2+^ flux entering the mitochondrial matrix facilitated by RaM is computed using equation 7, which is a modified form of the ion transport equation assuming a single, centered Eyring energy barrier and applying the Goldman constant field assumption [73]. We assume that RaM is a saturable transport mechanism [43] driven by the electro-chemical potential of Ca^2+^ across the inner mitochondrial membrane. In the presence of a physiological ΔΨ, the Ca^2+^ flux leaving the matrix via RaM is negligible. Therefore, to simplify model development and analysis, only the flux entering the matrix was considered. With these assumptions, the unidirectional, Ca^2+^ transport rate via RaM is computed as
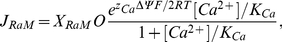
(7)where *X_RaM_* is the activity of RaM, *O* is the fraction of RaM in the open state, *z_Ca_* is the valence of Ca^2+^ (+2 unitless), *F* is Faraday's constant (96.480 J·mol^−1^·mV^−1^), *R* is the universal gas constant (8.314 J· mol^−1^·K^−1^), *T* is the temperature (298 K) and *K_Ca_* is the RaM affinity for Ca^2+^. Since ΔΨ was not reported in the data sets [42], [43], it was fixed at 190 mV for all the simulated experiments which corresponds to the typical experimentally determined value when mitochondria are “at rest” (state 2 respiration).

Since the only time dependent term in equation 7 is the fraction of RaM in the open state, it can be integrated from *t_0_* to *t* to obtain an analytical expression for the net Ca^2+^ uptake. This is calculated as
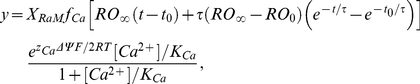
(8)where *y* is the computed Ca^2+^ uptake for a given Ca^2+^ pulse height and duration. Equation 8 was used when fitting the RaM parameters to the data sets characterizing RaM-facilitated Ca^2+^ uptake.

### Parameter Estimation and Analysis

The 4-state RaM model parameters were obtained by fitting the model simulated outputs to the available experimental data using the standard least squares method. The error function that was used measures the sum of the squared residuals weighted by the variance of the experimental data:
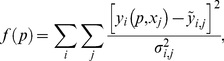
(9)where *f*(*p*) is the sum of the variance-weighted residuals evaluated at some parameter set *p*; 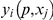 is the *i* ^th^ simulated output obtained with parameter set *p* for the *j* ^th^ experiment sample point corresponding to 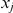; 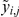 is the experimental measurement value corresponding to the *i* ^th^ simulated output at the *j* ^th^ experimental sample point with 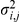 as the associated variance. The simulated Ca^2+^ uptake responses were obtained using equation 8 and used in equation 9 to simultaneously optimize the model parameters for each model.

The parameter covariance matrix was used to compute the confidence intervals. This matrix was estimated from the inverse of the Fisher Information Matrix (FIM). Often times the parameter covariance matrix is ill-conditioned due to large differences in parameter values or model outputs. This issue is primarily responsible for inaccurate approximations to the confidence intervals; therefore, the model outputs comprising the sensitivity matrix were normalized with respect to the best parameter value and experimental standard deviations. The normalized sensitivity matrix was computed as
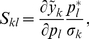
(10)where the normalized model output *ỹ_k_* is defined as *y_k_*/*σ_k_* where *k* consists of the indices of the concatenation of the *i*th simulated output at every sampling point, *l* is the index of the parameters and p^*^ is the best identified parameter value in terms of model fitness. The parameter covariance matrix was estimated using equation 10 and defined as
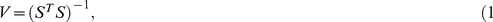
(11)


The confidence intervals computed from the parameter covariance matrix set the lower bound of the variance for a given parameter known as the Cramér-Rao Bound. Therefore, the 95% confidence intervals can be computed using equation 11 as shown as, 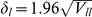, where *δ* is the normalized *l*th confidence interval. Thus, the absolute parameter confidence intervals for parameter *p_l_* is given by p_l_^*^(1± *δ_l_*). Note, that in most cases, the confidence intervals computed in this manner are an underestimation; however, they still give an accurate picture of the relative confidence in the model parameters.

### Corroboration Simulations

The data presented in Gunter et al. [14] was used to corroborate the RaM model. In this work, they utilized a novel method to deliver a pulsatile Ca^2+^ input to isolated mitochondria loaded with fluo-4, a Ca^2+^-sensitive fluorescent indicator. The mitochondria were attached to a coverslip where the response to a Ca^2+^ pulse from an individual, stationary mitochondrion could be monitored. The method used “caged Ca^2+^” or NP-EGTA. NP-EGTA is a strong Ca^2+^ chelating agent which is rapidly photodegraded upon exposure to UV. When degraded, the bound Ca^2+^ is released giving a rapid and localized increase in buffer Ca^2+^. Together with the rapid diffusion of Ca^2+^ away from the site of UV exposure, the method produces a pulse-like input for the isolated mitochondria.

The experimental system described above was reproduced *in silico* using a minimal, integrated model possessing the key components of the system in order to reduce the number of additional parameters required to simulate the dynamics. The following simplified dynamical system was used:
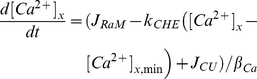
(12a)

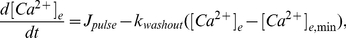
(12b)where [Ca^2+^]_x_ is the matrix free Ca^2+^ concentration, [Ca^2+^]_e_ is the buffer free Ca^2+^ concentration, *J_RaM_* is the rate of Ca^2+^ influx via RaM computed with the liver parameters found in Table 1 and equation 7 except that equations 1 and 2 were used to compute the fraction of RaM in state *O* since the external Ca^2+^ was not fixed, *k_CHE_* is a first order rate constant approximating Ca^2+^ release via the CHE, [Ca^2+^]*_x_*_,min_ is the basal matrix [Ca^2+^], *J_CU_* is the rate of Ca^2+^ influx via the CU, *β_Ca_* is the buffering capacity of the mitochondrial matrix, *J_pulse_* is a square wave simulating the Ca^2+^ release from the UV laser flash, *k_washout_* is the first order rate constant approximating Ca^2+^ diffusion away from the site of photodegredation and [Ca^2+^]*_e_*_,min_ is the basal buffer [Ca^2+^]. In the experimental protocol, Gunter et al. [14] reported that two different UV pulse durations were used to release the caged Ca^2+^: the experiment with the higher frequency used a pulse duration of 5.5 ms while the experiment with the lower frequency used a pulse duration of only 0.5 ms. Also, they reported that the 0.5 ms UV pulse gave a Ca^2+^ pulse with a peak of 400–500 nM [14]. Assuming linearity, the 5.5 ms UV pulse would yield a Ca^2+^ pulse with a peak of approximately 5 µM. These peak values were used when simulating the dynamical system presented in equation 12 and shown in Figure 4. Also, the diameter of the UV laser was assumed to be much larger than the size of a mitochondrion, so the Ca^2+^ exchanged between the buffer and mitochondrion was assumed to be negligible with respect to the buffer Ca^2+^. Thus, the mitochondrial uptake and release fluxes are not included in equation 12b. The rate of Ca^2+^ influx via the CU was approximated using the following expression taken from [3]:
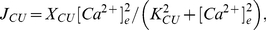
(13)where *X_CU_* is the approximate CU activity and *K_CU_* is the CU affinity for Ca^2+^. The parameter values for the simplified dynamical system is located in Table 2.

**Table 2:** Corroboration Model Parameters.

Parameter	Definition	Value	Units
*k_CHE_*	Ca^2+^ Extrusion Rate	350	s^−1^ a
[Ca^2+^]*_x_*_,min_	Basal Matrix Ca^2+^	90	nM^b^
*β_Ca_*	Matrix Ca^2+^ Buffering Strength	1000	unitless^c^
*k_washout_*	Rate of Ca^2+^ Unidirectional Diffusion	0.99	s^−1^
[Ca^2+^]*_e_*_,min_	Basal Buffer Ca^2+^	90	nM^d^
*X_CU_*	CU Activity	2×10^5^	nM s^−1^ e
*K_CU_*	CU Affinity for Ca^2+^	10	µM^f^

a Estimated from [74].

b Assuming electroneutral CHE and negligible ΔpH.

c Estimated from [75].

d Given by [14].

e Based on [3] and compensated for an appropriate ΔΨ.

f Estimated from [3].

### Supporting Information

In the File S1, a 5-state RaM model is derived to simulate the biphasic recovery observed in the heart RaM study. The 5-state RaM model is also compared to the 4-state RaM model with respect to the data, steady state behavior and recovery dynamics. The implications of the 5-state RaM model when subjected to an *in vivo*-like Ca^2+^ pulse train is then explored, and it is shown that a bimodal 4-state RaM model, where a small fraction is liver-like while the majority is heart-like, would be a far more relevant mode of Ca^2+^ uptake without compromising mitochondrial integrity.

## Supporting Information

File S1 **5-STATE RAM MODEL.**
(DOC)
