# Supplementary material for: A Minimal Model for the Mitochondrial Rapid Mode of Ca2+ Uptake Mechanism
Source: PLoS One. 2011 Jun 23;6(6):e21324. doi: 10.1371/journal.pone.0021324 (PMC3121760; doi:10.1371/journal.pone.0021324)
Supplement: File S1 — 5-STATE RAM MODEL. (DOC) [file pone.0021324.s001.doc]

**Supporting Information S1: 5-STATE RAM MODEL**

**5-STATE RAM MODEL DERIVATION**

The 5-state RaM model structure is similar to the 4-state RaM model, except that an additional slow recovery state was incorporated in order to reproduce the experimentally reported biphasic recovery dynamics of RaM . The state model diagram is pictured in Figure S1. This slow recovery state links the 2nd inhibited state to the rest state via an additional pathway. During an uptake cycle, about a quarter of the RaM transporters are reported to quickly reset while the remaining take up to 90 seconds to reset. This is interpreted as a large fraction of the RaM transporters entering a slow recovery state.


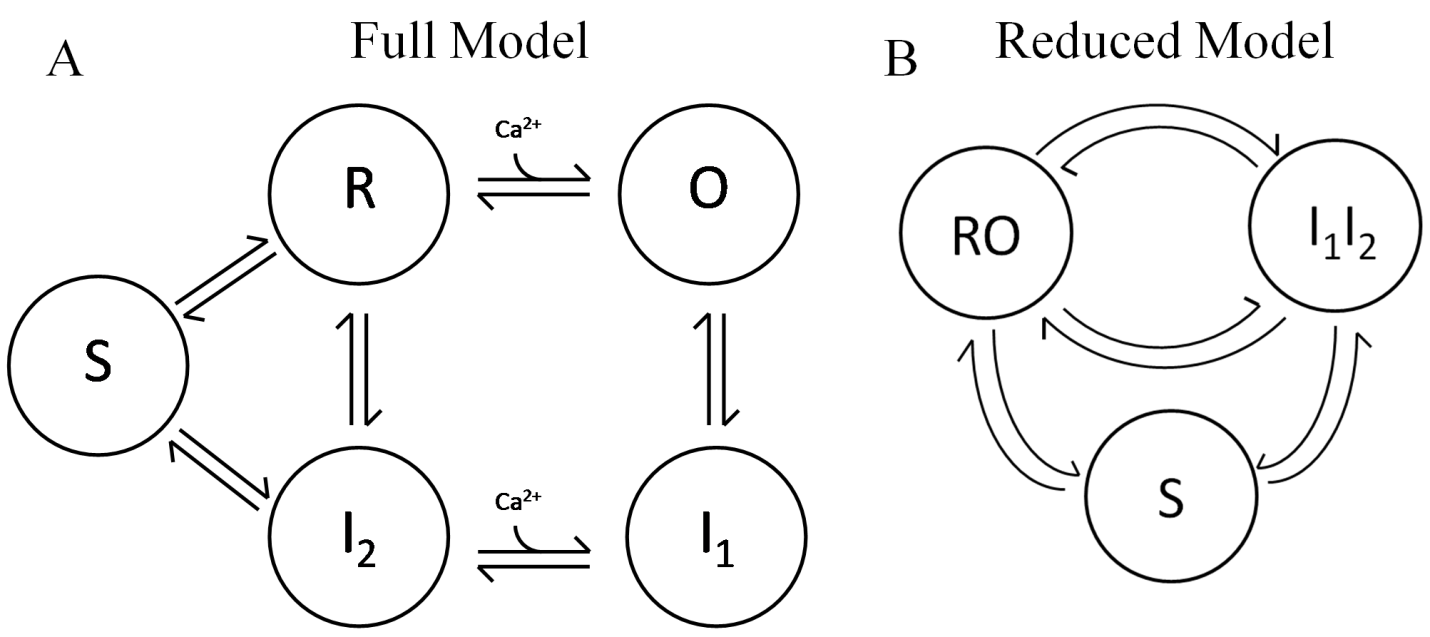


**Figure S1. The state model used to derive the 5-state RaM model for heart.** A) The full 5-state model is pictured where *R*, *O*, *I_1_*, *I_2_* and *S* represent the resting state, open state, 1st inhibited state, 2nd inhibited state and "slow" recovery state, respectively. B) For the reduced form, it is assumed that states *R* and *O* and *I_1_* and *I_2_* are in rapid equilibrium with each other. With this assumption, the 5-state model is reduced to a 3-state model where *RO* and *I_1_I_2_* represent the resting-open state and sum-total inhibited state, respectively, with the *S* state as previously defined. Applying conservation (i.e. *RO* + *I_1_I_2_* + *S* =1), the 3-state model is reduced to a set of ordinary differential-algebraic equations.

As with the 4-state RaM model derivation, the notation representing the explicit dependence on [Ca^2+^] for any expression defined below will be dropped (e.g. *f_Ca_*([Ca^2+^]) will be represented as *f_Ca_* as shown in equation S1a). The dynamics for the reduced 5-state RaM model are captured by the following set of deterministic ordinary differential-algebraic equations:

| $\begin{matrix} \frac{dRO}{dt}=k_{SR}S+\left[g_{Ca}k_{I_{1}O}+\left(1-g_{Ca}\right)k_{I_{2}R}\right]I_{1}I_{2}-\left[f_{Ca}k_{OI_{1}}+\left(1-f_{Ca}\right)\left(k_{RI_{2}}+k_{RS}\right)\right]RO \\ \frac{dI_{1}I_{2}}{dt}=k_{SI_{2}}S+\left[f_{Ca}k_{OI_{1}}+\left(1-f_{Ca}\right)k_{RI_{2}}\right]RO-\left[g_{Ca}k_{I_{1}O}+\left(1-g_{Ca}\right)\left(k_{I_{2}R}+k_{I_{2}S}\right)\right]I_{1}I_{2} \\ S=1-RO-I_{1}I_{2} \end{matrix}$ | (S1a),  (S1b),  (S1c), |
| --- | --- |

where *RO* represents the fraction of RaM in the rest and open states, *I_1_I_2_*  is the fraction of RaM in the inhibited states, S is the fraction of RaM in the slow recovery state, $k_{I_{1}O}$ is the transition rate from *I_1_* to *O*, $k_{I_{2}R}$is the transition rate from *I_2_* to *R*, $k_{OI_{1}}$ is the transition rate from *O* to *I_1_*, $k_{RI_{2}}$is the transition rate from *R* to *I_2_*, $k_{RS}$is the transition rate from *R* to *S*, $k_{SR}$ is the transition rate from *S* to *R*, $k_{I_{2}S}$is the transition from *I_2_* to *S*, $k_{SI_{2}}$ is the transition from *S* to *I_2_*, *f_Ca_* is the fraction of the state *RO* with Ca^2+^ bound and *g_Ca_* is the fraction of state *I_1_I_2_* with Ca^2+^ bound. The relationship between the rest and open states as a function of Ca^2+^ is expressed as

| $f_{Ca}=\frac{[Ca^{2+}]^{n}}{[Ca^{2+}]^{n}+K_{O}^{n}}$ | (S2), |
| --- | --- |

where *K_O_* is the threshold concentration that triggers the cycling mechanism and *n* is the Hill coefficient representing the steepness of the profile for the fraction of bound versus free RaM. Therefore, the amount of RaM in the rest or open states can be computed using $R=\left(1-f_{Ca}\right)RO$ and $O=f_{Ca}RO$, respectively. The relationship between the first and second inhibited states is similarly computed as

| $g_{Ca}=\frac{[Ca^{2+}]^{n}}{[Ca^{2+}]^{n}+K_{I}^{n}}$ | (S3), |
| --- | --- |

where *K_I_* is the threshold concentration locking the cycling mechanism in the inhibited state, and *n* is the Hill coefficient representing the steepness of the profile for the fraction of bound versus free RaM for the inhibited states. The fraction of inhibited RaM without and with Ca^2+^ bound are computed as similar to the fraction of rest and open RaM states where $I_{1}=\left(1-g_{Ca}\right)I_{1}I_{2}$ and $I_{2}=g_{Ca}I_{1}I_{2}$, respectively.

As with the 4-state model, when the external [Ca^2+^] is fixed, the 5-state model (equation S1) becomes linear and can be integrated to obtain an analytical solution. The major difference between the analytical solutions is that the 5-state analytical solution involves a matrix exponential as shown below:

|  | (S4a),  (S4b),  (S4c),  (S4d), |
| --- | --- |

where *A* is the constant state-transition matrix (for fixed Ca^2+^), *I* is the identity matrix, *b* is the constant input vector derived by applying conservation between states *RO*, *I_1_I_2_* and *S* (i.e., *RO* + *I_1_I_2_* + *S* = 1), $RO_{0}$ is the initial value of state *RO* and is the initial value of state *I_1_I_2_*.

Since the 5-state model posses biphasic dynamics separated by more than two orders of magnitude, the time constants can be approximated using the following analytical expressions:

| $\begin{matrix} \tau_{f}=1/\left[f_{Ca}k_{OI_{1}}+\left(1-g_{Ca}\right)k_{I_{2}R}+\left(1-f_{Ca}\right)k_{RI_{2}}+g_{Ca}k_{I_{1}O}\right] \\ \tau_{s}=1/\left[k_{SR}+\left(1-f_{Ca}\right)k_{RS}\right] \end{matrix}$ | (S5a),  (S5b), |
| --- | --- |

where *τ_f_* and *τ_s_* are the fast and slow time constants, respectively. The steady state fraction of state *RO* is given by:

| $RO_{\infty}=-\left[\begin{matrix} 1 & 0 \end{matrix}\right]A^{-1}b$ | (S6). |
| --- | --- |

**Ca^2+^ uptake equation**

The Ca^2+^ uptake predicted by the reduced 5-state model was computed using a slightly different expression from equation 8. The uptake is computed by integrating equation S4a from *t_0_* to *t* similar to the 4-state model to obtain the following Ca^2+^ uptake expression:

|  | (S7). |
| --- | --- |

The 5-state model introduces four additional parameters ($k_{I_{2}S}$,$k_{SI_{2}}$,$k_{SR}$ and $k_{RS}$) relative to the 4-state model. As with the 4-state model, one of these parameters is dependent on the others when microscopic reversibility is enforced; therefore, the 5-state model required nine adjustable parameters.

**Parameter Estimation and Analysis**

The model parameters were obtained by fitting the model to the available experimental data using the method outlined in Material and Methods. The results are presented below in Table S1. In brief, the simulated Ca^2+^ uptake responses were obtained using equation S7 and used in equation 9 to simultaneously optimize the model parameters for each model. The liver model fitting using the 4-state model was included when fitting the 5-state model since some model parameters were shared. The information encoded in the liver RaM experimental data help to improve parameter identifiability.

**Table S1.** 5-State RaM Model Parameters

| **Parameter** | **Definition** | **Value (Confidence Interval)** | **Units** |
| --- | --- | --- | --- |
|  |  | 5 State |  |
| *X_RaM_* | RaM Activity | 0.0551 (0.0546-0.0557) | nmol mg^-1^s^-1^ |
| *K_Ca_* | RaM Complex Ca^2+^ Affinity | 340 (331-348) | nM |
| *n* | Ca^2+^ Cooperativity | 24 | unitless^a^ |
| *K_O_* | State O Ca^2+^ Binding Constant | 224 (223-224) | nM |
| *K_I_* | State I_1_ Ca^2+^ Binding Constant | 110 (109-110) | nM |
| $k_{OI_{1}}$ | State O-I_1_ Transition Rate | 100 | s^-1b^ |
| $k_{I_{1}O}$ | State I_1_-O Transition Rate | 0.0364 | s^-1c^ |
| $k_{I_{2}R}$ | State I_2_-R Transition Rate | 1 (0.001-6.96) | s^-1^ |
| $k_{RI_{2}}$ | State R-I_2_ Transition Rate | 1.04x10^-5^ (1.04x10^-7^-4.340x10^-4^) | s^-1d^ |
| $k_{I_{2}S}$ | State I_2_-S Transition Rate | 5.36 (0.0054-34.5) | s^-1d^ |
| $k_{SI_{2}}$ | State S-I_2_ Transition Rate | 0.0229 | s^-1a^ |
| $k_{SR}$ | State S-R Transition Rate | 0.0332 (0.0234-0.0409) | s^-1^ |
| $k_{RS}$ | State R-S Transition Rate | 7.81x10^-4^ | s^-1c^ |

^a^Removed from confidence interval analysis due to unidentifiability. In this case, during the confidence interval estimations, the parameters were fixed to the values as shown.  ^b^Fixed to the upper bound time constant of RaM in the open state . ^c^Set by enforcing microscopic reversibility where $k_{I_{1}O}=k_{OI_{1}}k_{I_{2}R}K_{I}^{n}/k_{RI_{2}}/K_{O}^{n}$ and $k_{RS}=k_{SR}k_{RI_{2}}k_{I_{2}S}/k_{SI_{2}}/k_{I_{2}R}$.  ^d^When the estimated lower bound was less than 0, it was set to 10^-3^ of the best parameter value.

**RESULTS AND DISCUSSION**

Using the heart parameters listed in Tables 1 and S1, the model simulations for the 4- and 5-state models compared to the experimental data for heart RaM is presented in Figure S2. The models were all able to reproduce the Ca^2+^ uptake profiles for a sustained Ca^2+^ pulse (Figure S2A), varied interpulse durations (Figure S2B), varied interpulse heights (Figure S2C) and the increases in Ca^2+^ uptake efficiency for multiple pulses versus a single, sustained pulse (Figure S2D). Both were capable of fitting the heart data with near identical quality; only minor quantitative differences are seen between each model.

**
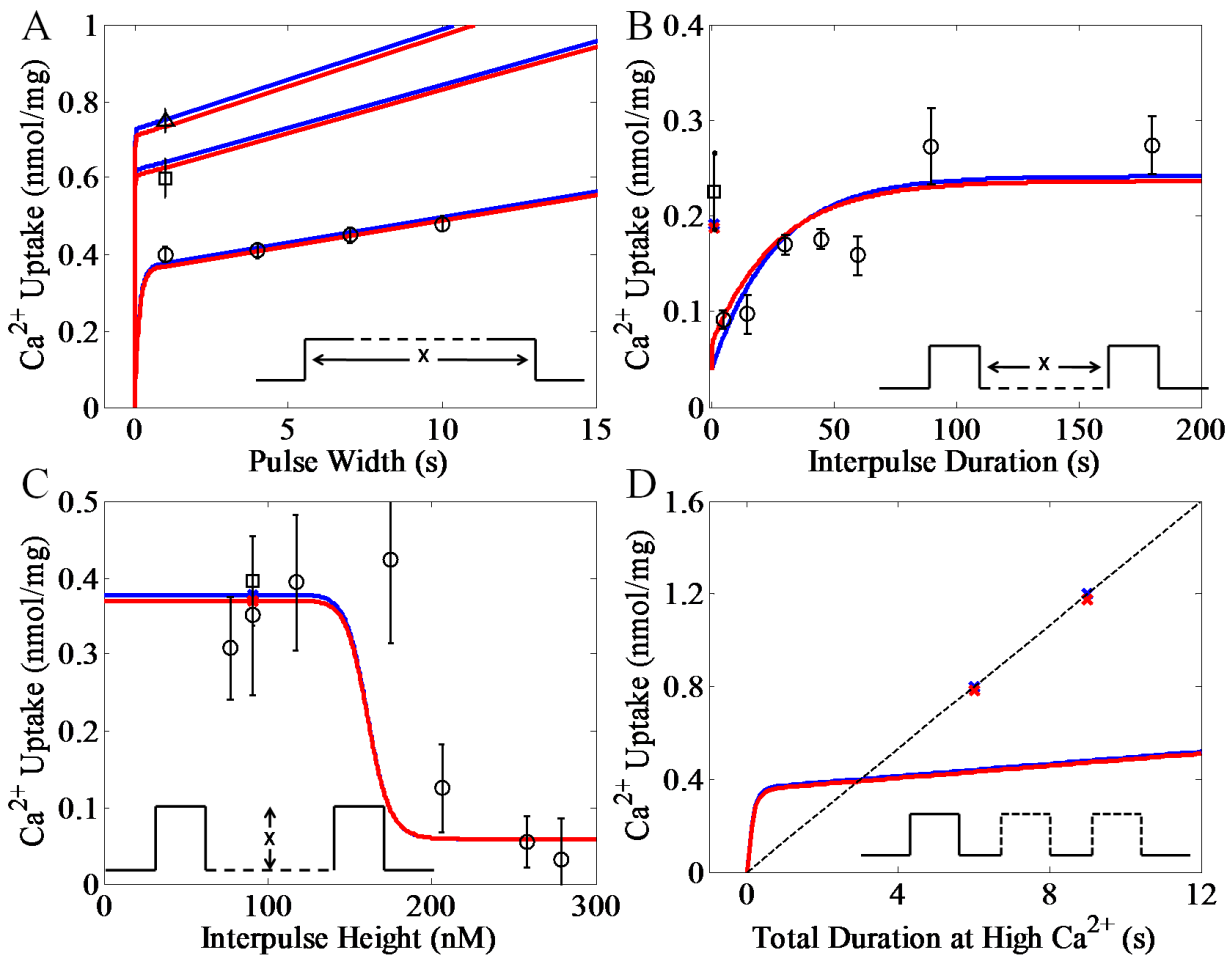
**

**Figure S2. Simulations of the experimental data of Ca^2+^ uptake facilitated by RaM in heart.** All models were able to reproduce the major features of RaM presented in the experimental literature. The blue and red lines correspond to simulations of the 4- and 5-state models, respectively. The corresponding pulse waveform for each experiment is shown in each panel. A) The model reproduced the measured Ca^2+^ uptake responses to pulses of Ca^2+^ at varying pulse heights and durations. The circles represent the Ca^2+^ uptake from a single pulse at a pulse height of 201 nM with an increasing pulse duration. The square and triangle represent the Ca^2+^ uptake from a 1 s duration Ca^2+^ pulse at a pulse height of 567 and 934 nM, respectively. The prepulse height for each pulse was 53 nM. B) The model reproduced the measured Ca^2+^ uptake response to a second pulse of Ca^2+^ following a previous pulse of the same magnitude at varying interpulse durations. The circles represent the Ca^2+^ uptake from a second pulse immediately after and up to 180 s after the first pulse. Both pulses were 5 s in duration at a pulse height of 209 nM with an interpulse height of 98 nM. The square represents a single, 5 s pulse at a pulse height of 181 nM with a prepulse height of 86 nM. C) The model reproduced the measured Ca^2+^ uptake response to a second pulse of Ca^2+^ following a previous pulse of the same magnitude at varying interpulse heights at a fixed interpulse duration of 60 s. Each pulse duration was 5 s. The circles represent the Ca^2+^ uptake from the second pulse at a pulse height of 274 nM. The square represents a single pulse at a pulse height of 273 nM for a duration of 5 s. D) The model reproduced the enhanced Ca^2+^ uptake response to multiple, short pulses versus a single, long pulse. The dashed line follows the trend of increasing Ca^2+^ uptake for in an increasing the number of pulses. All simulation results are either shown as x's or a solid line depending on the nature of the simulation. The only major difference between the 4-state and 5-state models is shown in B) where the 5-state model possesses a biphasic recovery of two different time scales.

Figure S3 demonstrates that the steady state *R* state values are nearly identical with the time constants differing significantly when [Ca^2+^] drops below the *K_I_*. It also shows that the 2^nd^ time constant, the "slow" time constant, for the 5-state model is nearly independent of [Ca^2+^].

**
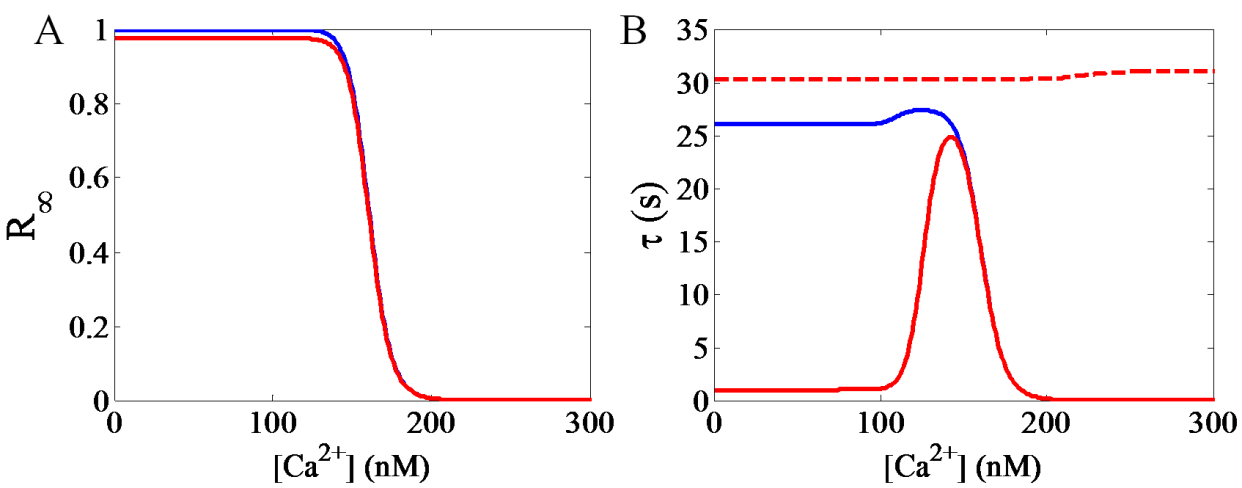
**

**Figure S3. The steady state values for state *R* and time constants for the models as a function of Ca^2+^ is presented for the 4- (blue) and 5-state (red) heart RaM.** A) The steady state values for state *R* are identical for the 4-state and 5-state models. B) The "fast" time constant (solid) is identical when [Ca^2+^] exceeds the *K_O_* (224 nM); however, they diverge when [Ca^2+^] drops below the *K_I_* (110 nM). The "slow" time constant (dash) for the 5-state model is nearly constant regardless of [Ca^2+^].

The differences between the recovery profiles for the 4- and 5-state models parameterized with the heart RaM parameters as a function of [Ca^2+^] is compared in Figure S4. The 4-state model recovers with a single time constant while the 5-state model shows a biphasic recovery as suggested by the experimental data . The inset of Figure S3B shows the fast recovery in more detail. Note that it is near complete by 400 ms similar to the fast recovery of liver RaM.


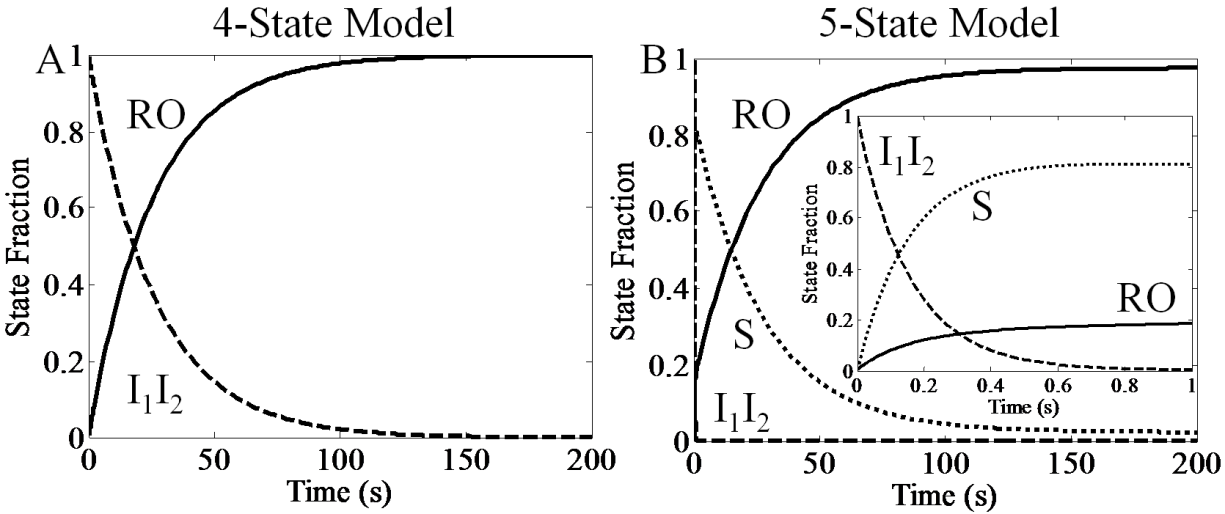


**Figure S4. The recovery profile of the 4- and 5-state models when external** [Ca^2+^] **is dropped below 100 nM.** A) The 4-state model possess a single time constant of approximately 25 seconds to recover when the external [Ca^2+^] was set to 50 nM. B) The 5-state model shows a biphasic recovery and thus possesses two time constants when the external [Ca^2+^] was set to 50 nM. The time constant is approximately 25 seconds to recover from the *S* state, while it is only about 0.1 seconds to recover from the *I_2_* state; however, approximately 75% of RaM enters the slow recovery state while the remaining 25% is quickly reset. The fast resetting dynamics are shown in the inset of plot B. The trajectories for the states *RO*, *I_1_I_2_* and *S* are represented by the solid, dashed and dotted lines, respectively. For clarity, the state trajectories are identified in each plot. Note that the *S* state is absent for the 4-state model simulations. The model was initially in the fully inhibited state.

There is evidence that heart RaM is rapidly reset despite what was previously reported. Rat heart mitochondria subjected to a Ca^2+^ pulse train displayed rapid Ca^2+^ uptake with a period of only 6 seconds . Moreover, the matrix Ca^2+^ appeared to increase in discrete quantities per pulse until external Ca^2+^ increased above a critical concentration. This behavior is indicative of RaM. The mitochondria used in the heart RaM study were isolated from 4 week old White Leghorn chicks , so the species, as well as, developmental stage differences may be responsible for the apparent discrepancy between resetting times for RaM. Also, in the heart RaM study, Buntinas et al. never reported the Ca^2+^ uptake profile for an experiment with a Ca^2+^ pulse train administered at a near physiological frequency . So it is unknown if the heart RaM actually possess an additional slow state or not. Buntinas et al. concluded that despite the slow recovery of heart RaM, the uptake of a second pulse immediately after the first pulse is capable of sequestering up to 25% of the first pulse. Thus the biphasic recovery appeared to be a reasonable mechanism behind this phenomenon; however, Figure S5 demonstrates that a single type of RaM that possesses this biphasic recovery would be essentially non-functional in heart. It would thus be more appropriate for two types of RaM to exist in heart, one with the fast recovery exhibited by the liver-type and another with the slow recovery exhibited by the heart-type. This bimodal functionality would be able to explain some of the kinetic curiosities discovered about heart RaM and still be consistent with the available experimental data . Moreover, it seems doubtful that evolution would preserve such a unique Ca^2+^ transporter that would be essentially inert in heart mitochondria. Unfortunately, this issue will not be resolved until further experimental evidence that characterizes the resetting dynamics for heart RaM is collected.

The simplified dynamical model of mitochondrial Ca^2+^ handling presented in the Material and Methods was simulated to produce the results shown in Figure S5. For the simulations only considering the 5-state RaM model (A and B), the matrix [Ca^2+^] initially reaches approximately 1 µM upon the beginning of the pulse train but is quickly depleted as more and more of the fraction of RaM accumulates in the *S* state. For the simulations exploring the bimodal hypothesis (C and D), the matrix [Ca^2+^] is maintained due to the rapid recover of the liver-like RaM component while the heart-like RaM component remains essentially inactivated. In the simulations shown in Figure S5C and S5D, the 4-state models described in the main manuscript were used except that the activities for the heart-type and liver-type were 75% and 25% of *X_RaM_* for heart given in Table 1, respectively.


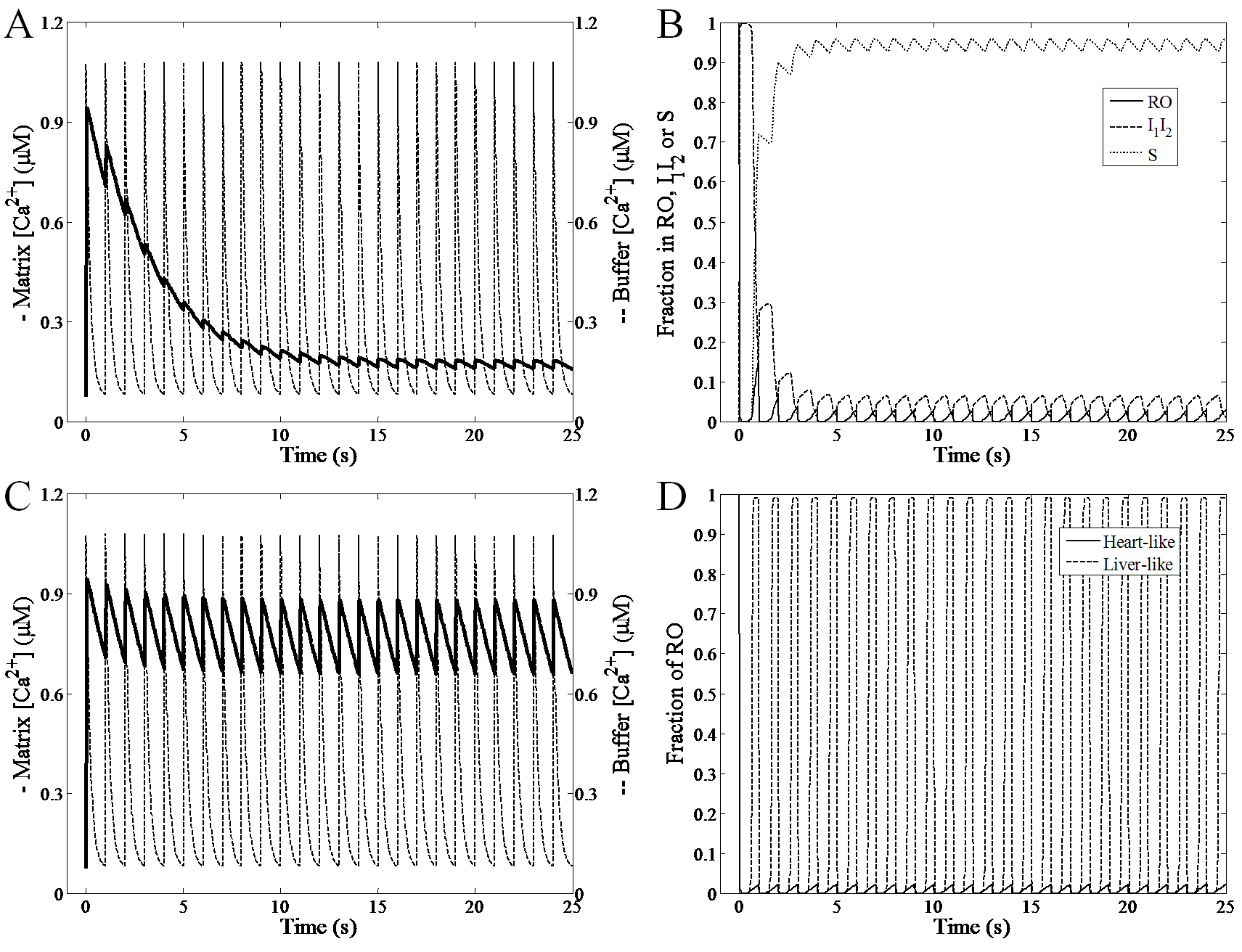


**Figure S5. In heart, the bimodal RaM model would alleviate the slow-recovery phenomenon observed with the 5-state model.** The Ca^2+^ fluxes for the 5-state or bimodal RaM models were used in place of *J_RaM_* for the simplified Ca^2+^ dynamics model given in the Material and Methods. A) The matrix and buffer Ca^2+^ dynamics given by the 5-state RaM model with a 1 µM/s Ca^2+^ pulse train is shown. B) The corresponding fraction of RaM in either the *RO*, *I_1_I_2_* or *S* state is shown. C) The matrix and buffer Ca^2+^ dynamics given by the bimodal RaM model with a 1 µM/s Ca^2+^ pulse train is shown. D) The corresponding fraction of RaM in the *RO* state for the heart-like and liver-like RaM transporters is shown.

**REFERENCES**

1. Buntinas, L., K. K. Gunter, G. C. Sparagna, and T. E. Gunter. 2001. The rapid mode of calcium uptake into heart mitochondria (RaM): comparison to RaM in liver mitochondria. Biochim Biophys Acta 1504:248-261.

2. Gunter, T. E., D. I. Yule, K. K. Gunter, R. A. Eliseev, and J. D. Salter. 2004. Calcium and mitochondria. FEBS Lett 567:96-102.

3. Beutner, G., V. K. Sharma, D. R. Giovannucci, D. I. Yule, and S. S. Sheu. 2001. Identification of a ryanodine receptor in rat heart mitochondria. J Biol Chem 276:21482-21488.
